# Bridging the gap in the UK’s National Health Service integrated care systems: insights from a mixed methods implementation evaluation of UCLP-PRIMROSE, a care innovation to reduce physical health inequalities for people with severe mental illness

**DOI:** 10.1136/bmjopen-2025-105511

**Published:** 2026-01-27

**Authors:** Philippa Shaw, Zuneera Khurshid, Danielle Lamb, Fiona A Stevenson, Gregor Russell, Kristian Hudson, Nirandeep Rehill, Gemma Copsey, Matt Kearney, Edward Beveridge, Ian Prenelle, David Osborn

**Affiliations:** 1 Division of Psychiatry, University College London, London, England, UK; 2 Improvement Academy, Bradford Institute for Health Research, Bradford, England, UK; 3 Department of Primary Care and Population Health, University College London, London, England, UK; 4 Primary Care and Population Health, Royal Free Campus, University College London, London, England, UK; 5 Bradford District Care NHS Foundation Trust, Saltaire, England, UK; 6 UCLPartners, London, England, UK; 7 North London NHS Foundation Trust, London, UK; 8 Barnet Enfield and Haringey Mental Health NHS Trust, London, England, UK

**Keywords:** Schizophrenia & psychotic disorders, Cardiovascular Disease, Implementation Science, Primary Health Care

## Abstract

**Abstract:**

**Objective:**

We aimed to determine whether UCLP-PRIMROSE (a care innovation to reduce physical health inequalities for people with severe mental illness) could be set up in the current UK National Health Service (NHS) context and identify the processes, barriers and facilitators to implementation.

**Design:**

We employed a convergent mixed methods approach, combining interviews, ethnographic site visits and the collection of meeting notes and uptake data for core model components. Interview transcripts were analysed using reflexive thematic analysis, and all qualitative data, including interview transcripts, were analysed using the Consolidated Framework for Implementation Research. Qualitative work and insights from implementation uptake frequencies were integrated using Normalisation Process Theory.

**Setting:**

We evaluated implementation in Yorkshire and three London boroughs, mainly within general practices.

**Participants:**

We conducted interviews with 39 staff members who were implementing and/or delivering UCLP-PRIMROSE.

**Intervention:**

UCLP-PRIMROSE is an integrated evidence-based care pathway developed to reduce cardiovascular disease risk and mental health relapse in people with severe mental illness.

**Results:**

Adaptation and delivery varied in completeness and consistency across 24 general practices and their wider care teams. Factors outside the implementation teams’ influence challenged the embedding of UCLP-PRIMROSE. Factors included the impact from the immaturity of NHS integrated care systems, unintended consequences of the incentivised NHS severe mental illness physical health check and limited capacity for implementing in a system facing resourcing challenges. Drivers of successful implementation included staff being aligned with the values of the UCLP-PRIMROSE model and system leaders acting as champions. Supportive foundational processes acted as facilitators: these included protected and prioritised time for reflection, learning and problem solving.

**Conclusions:**

Implementation of UCLP-PRIMROSE was moderately successful in a relatively short period of time. At the end of the research, all teams wanted to sustain delivery. However, further pathway simplification and additional resources are required to spread UCLP-PRIMROSE beyond early pockets of good practice.

**STRENGTHS AND LIMITATIONS OF THIS STUDY**

- We used mixed methods to explore the implementation of a new care pathway for people with severe mental illness at risk of cardiovascular disease, identifying barriers and facilitators relevant to wider cross-sector care and the management of multiple long-term conditions.
- We collected in-depth data and used complementary approaches to analysis.
- Our research team was interdisciplinary, and we collaborated with patient and public involvement groups from the design phase through to the creation of public-facing outputs.
- However, patient engagement in interview recruitment was limited, and there were gaps in the availability of quantitative data.

## Background

 There is a well-evidenced association between severe mental illness (SMI; such as schizophrenia and bipolar disorder) and poor physical health due to multiple factors, including the adverse impact of antipsychotic medications. Compared with the general population, people with SMI have a higher risk of cardiovascular disease (CVD).^1 2^ This is known to lead to a ‘mortality gap’, where people with SMI die 15–20 years earlier than the general population.^3^,^5^

Consequently, significant directives in UK policy to improve the health of this population have been published.^6^,^8^ This includes the incentivisation of health screening as part of a yearly annual physical health check provided for people with SMI under the Quality and Outcomes Framework (QOF), a voluntary reward and incentive programme for general practices.^8^ Yet, new reports suggest the mortality gap is worsening. In 2023, the Office for Health Improvement and Disparities reported a substantial excess of deaths in people with SMI in the UK from the previous year, highlighting several causes including CVD and diabetes.^9^ Reports such as the Lancet Psychiatry Commission into protecting physical health in people with mental illness state that those with SMI are still less likely to receive the care they need, particularly for long-term physical health conditions.^10^

While there continues to be mixed findings related to physical health outcomes across clinical trials examining lifestyle interventions for people with SMI, there is greater certainty of the effective components of lifestyle interventions and their value.^11^ Recent work has also suggested the benefit of multidisciplinary approaches, including the potential to increase engagement with such interventions.^11 12^

As the NHS moved to integrated care systems (ICSs) and more recently to include neighbourhood health services,^13^ the aim has been to bring together teams of health professionals to support people with multiple long-term conditions and complex needs. The ICSs are collaborative partnerships between NHS bodies, local authorities and community organisations operating through statutory integrated care boards and integrated care partnerships to improve health and wellbeing at a population level.^14^ Following Lord Darzi’s review of the NHS, the NHS 10-year plan was published, outlining guidance for integrated care boards.^15 16^ One of the three radical shifts is hospital to community through the development of neighbourhood health services, designed to integrate all parts of the system to improve population health.

We need to build on existing evidence to better understand how evidence-based care for people with SMI that integrates expertise (physical, mental and social) from across the system can be appropriately implemented. One approach to the care of people with SMI, which includes lifestyle intervention, is UCLPartners-PRIMROSE.

### UCLPartners-PRIMROSE

**Figure 1:**
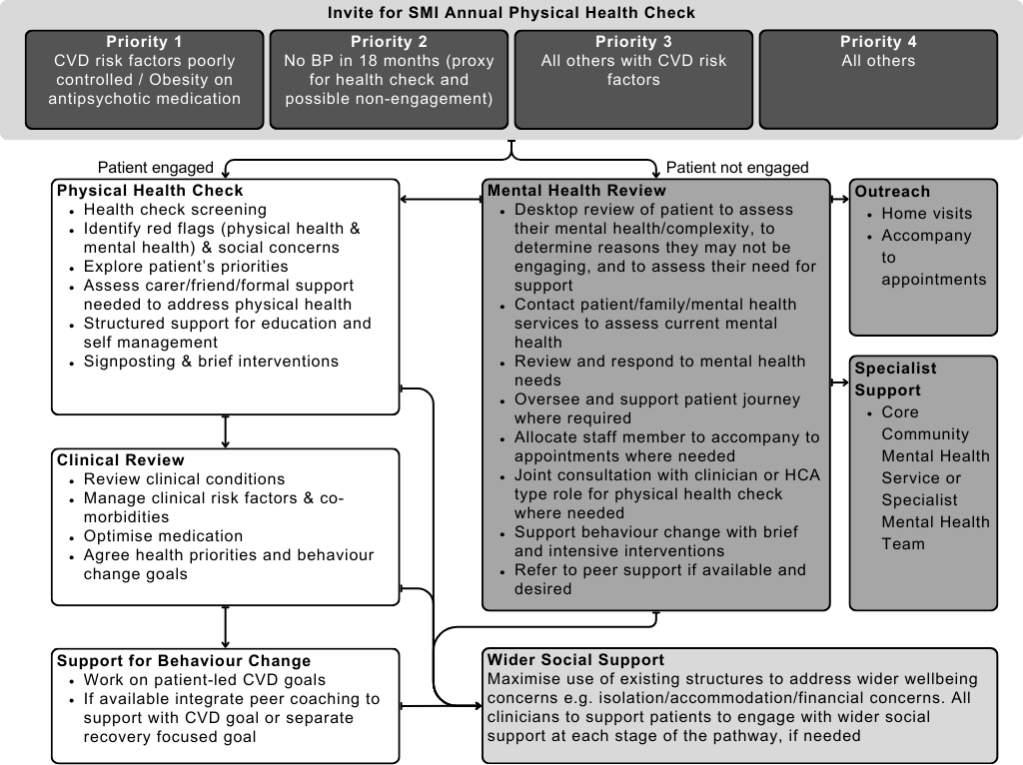
UCLPartners-PRIMROSE pathway. (BP, blood pressure; CVD, cardiovascular disease; HCA, health care assistant; SMI, severe mental illness.) The five core elements are: (1) Search and risk stratification tool (subsequently referred to as the search stratification tool) used to inform order of patient invitation for their SMI annual physical health check, (2) Provision of the physical health check, (3) Clinical review of the results from the health check and formation of a care plan, (4) Delivery of the care plan, and (5) Provision of support for patients who are not engaging with their health check or subsequent care.

UCLPartners-PRIMROSE or UCLP-PRIMROSE^17^ is the combination of two innovations: PRIMROSE-A^18,21^ and the UCLPartners Proactive Care Frameworks.^22^ See figure 1 for the resulting pathway. This was designed so that people with SMI receive medication optimisation (e.g., statin prescribing where necessary), structured support for education and self-management, holistic support for wider health and social needs, and those with the greatest CVD risk are prioritised. UCLP-PRIMROSE was designed to be delivered across primary and secondary care, involving the voluntary sector. See online supplemental additional file 1 for further details on the clinical model.^23^

UCLP-PRIMROSE incorporates several key components of effective lifestyle interventions identified by the third Lancet Psychiatry physical health commission, including a theoretical foundation, use of behaviour change techniques, and a structured approach.^11^ UCLP-PRIMORSE was developed with people with lived experience and includes peer delivery, features which were present in only 6% of interventions reviewed in the Lancet Psychiatry commission.^11^

### The current research

We evaluated the implementation of UCLP-PRIMROSE in Yorkshire and London. We aimed to investigate whether UCLP-PRIMROSE could be set up and delivered as part of service transformation (improvement to clinical care outside of controlled research like clinical trials) and identify the related facilitators, barriers and processes.

## Methods

We took a mixed methods approach, convergent parallel design, to understand the current context and the implementation of an integrated mental and physical health pathway across system boundaries.

We received a favourable opinion from NHS Research Ethics Committee (reference: 20/WS/0153; IRAS: 285554) and used the Standards for Reporting Qualitative Research to ensure comprehensive reporting^24^ (online supplemental additional file 2).

### Data collection

From March 2022 to August 2024, we conducted interviews, collated documents related to processes (e.g., notes from meetings and ethnographic site visits) and collected data related to UCLP-PRIMROSE uptake.

#### Interviews

We invited staff involved with the implementation and/or delivery of UCLP-PRIMROSE to take part in interviews by email. Interviews took place at baseline (before or near the start of delivery) and approximately 9 months later.

The two-stage interview schedule (baseline and ~9 months into delivery) was selected to align with real-world implementation rather than a trial cycle. The ~9-month follow-up provided enough time for UCLP-PRIMROSE delivery activity to begin, included site preference to start delivery with the new QOF year and allowed staff to reflect on barriers, facilitators and emerging consequences while remaining within the research timeframe. This timing also accommodated staggered onboarding and role changes across sites, meaning different professional groups engaged with the pathway at different points.

Participants were given the option for interviews (in-person, phone or online). These options were designed for flexibility. All interviews took place online via Microsoft Teams, except one (conducted in person).

Topic guides (onlinesupplemental additional file 3  4) were developed with reference to the Consolidated Framework for Implementation Research (CFIR), focusing on open-ended prompts about what happened in practice, perceived barriers and perceived enablers. Interviews were semistructured: the research team had the CFIR domains in front of them as prompts and could introduce questions from any domain that had not yet been covered, ensuring relevant areas were explored while allowing participants to lead the discussion.

We conducted 46 interviews with 39 staff members: baseline only (n=21), ~9 months only (n=11) and both baseline and at ~9 months (n=7). Interviews were recorded in Microsoft Teams, transcribed verbatim and deidentified by PS and ZK.

Staff delivering UCLP-PRIMROSE discussed the research with people who had received care via the pathway and asked their permission to pass their contact details to the researcher for an interview. No patients consented to interview (preferring to provide views informally via patient feedback via phone call); therefore, no patient qualitative data are reported in this paper.

#### Process data

We collected 214 documents related to implementation, including meeting video recordings and minutes, notes from training sessions and implementation plans. Additionally, we collected 10 documents of field notes from ethnographic site visits and check-ins with staff and created 14 lightning reports;^25^ short reports structured as ‘what works’, ‘what needs to be changed’ and ‘insights, ideas and recommendations’ to summarise ongoing learning. We subsequently refer to this data collectively as process data.

#### Implementation uptake data

As the purpose of this research was to investigate implementation rather than assess UCLP-PRIMROSE effectiveness, we collected data around pathway use from seven case study sites. These sites were the first general practices engaged with the research across the four locations. In site 4, implementation was taking place at the primary care network level; therefore, all four practices were included.

We collected information about the delivery of the pathway, including model variation (such as staff members involved) and uptake data (e.g., how many patients had received a clinical review). For the latter, we either extracted data from patient records, reviewed spreadsheets used by some staff to track patient care or received summary information from project teams.

#### Sample size

We acknowledge the broad and variable recommendations regarding sample size for participants. For example, compared with some recommendations, we engaged with a number of interview participants at the larger end of the scale.^26^ However, we approached the justification of our sample size with a critical understanding of the concept of saturation, particularly with qualitative research, as discussed by Braun and Clarke in relation to thematic analysis.^27^ Across the multiple methods used, we aimed instead for depth and breadth over quantity, with purposeful recruitment and complementary data sources, including process data from over 200 hours of meetings. We deemed this reasonable and substantial when we approached integration.

### Data analysis

We used two analytic frameworks for our qualitative data, reflexive thematic analysis^28 29^ and CFIR^30^, and explored data around the uptake of UCLP-PRIMROSE through frequencies. We integrated our findings using Normalisation Process Theory (NPT).^31 32^

#### Rationale

We selected to use reflexive thematic analysis to explore the interview transcripts without the limits of predefined constructs. This was appropriate given the exploratory nature of our research, investigating if implementation was possible and gaining insight into facilitators, barriers and processes.

Our analytic strategy also combined a determinant framework (CFIR) with a process theory (NPT). CFIR was used to characterise contextual determinants (for example, resources, leadership, organisational culture and system-level enablers and constraints) at the site level. NPT was then applied to interrogate the ‘work’ required to embed UCLP-PRIMROSE in everyday practice (with constructs organised under context, mechanism and outcome).^32^ We treated these as complementary rather than redundant: CFIR helped us explain *what* conditions supported or hindered implementation; NPT helped us explain *how* people were actively trying to make the pathway routine despite those conditions.

#### Reflexive thematic analysis

Our work was guided by one way of conducting thematic analysis, the procedures outlined by Braun and Clarke *et al*.^28^ We iteratively followed the steps of familiarisation with the interview transcripts, initial line-by-line coding using NVivo V.20^33^, generating themes, reviewing the themes and creating a theme map, defining and naming themes and writing up.

#### Consolidated framework for implementation research

We used CFIR 1.0 to explore context‐dependent factors impacting implementation.^30^ We used the original CFIR rather than CFIR 2.0, as our work began before the release of CFIR 2.0^34^. The CFIR defines five domains (intervention characteristics, outer setting, inner setting, characteristics of individuals and process), each with subconstructs which can affect implementation success.

For the interview transcripts, two different applications of the CFIR were used. PS applied CFIR line by line in NVivo, whereas ZK took the subthemes from the reflexive thematic analysis and mapped these onto CFIR subconstructs. For the process data, PS and ZK applied CFIR line by line.

We then collated CFIR-coded data, by site and by collected data type, into memos (Word documents including ratings of construct impact on implementation, summaries and rationales). All ratings were then collated into a matrix in Excel.

#### Quantitative data

We explored uptake data through frequencies, for example, the proportion of eligible patients receiving UCLP-PRIMROSE-related care through each element of the pathway. When not provided by implementation teams, these were calculated through breaking down extracted patient data into frequencies using Excel (V.2410).

### Integration across findings

Our approach to endpoint integration involved multiple stages and was informed by previous research.^35 36^

After coding, we produced master integration tables linking (1) reflexive thematic codes, (2) CFIR constructs, (3) illustrative excerpts, (4) understanding from uptake data and (5) NPT coding manual constructs.^32^ A condensed version of our initial mapping of CFIR, NPT and reflexive thematic codes is provided in online supplemental additional file 5.

Our integration was subsequently developed through diagrams, theme creation and the write-up. We met with members of the wider research team at several points during the study to discuss findings from the different approaches to analysis and finding integration.

### Patient and public involvement

We worked with two patient and public involvement groups (The DIAMONDS Patient and Public Engagement Panel (DIAMONDS Voice) and the Yorkshire Quality and Safety Patient Panel) to develop our research procedures and materials. They informed research processes (such as the consideration of when to inform patients about the research and reasonable adjustments) and topic guide content. We also collaborated with DIAMONDS Voice to explore and interpret our findings and co-develop outputs to share with public audiences, including service users and carers.

## Results

**Figure 2:**
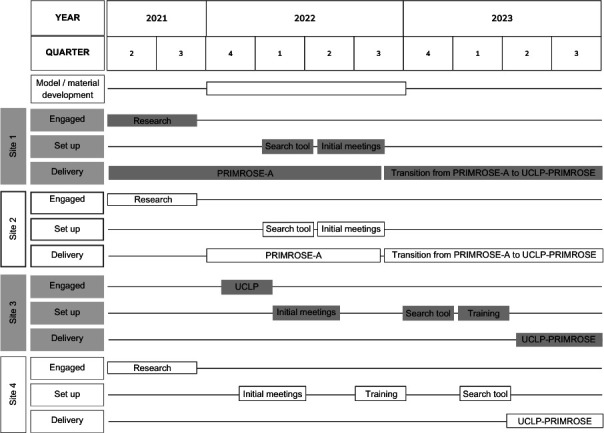
Diagram illustrating the broad timeline of development of UCLP-PRIMROSE and onboarding of sites. The timeline is presented against the NHS financial year as this was the calendar implementation and delivery teams worked against. Included in the timeline is the onboarding of sites, with sites 1, 2, and 4 onboarded at research development and grant application stage, whereas site 3 was engaged later by Health Innovation Network UCLPartners. ‘Search tool’ refers to first importing of the search stratification tool into local electronic patient record systems and ‘initial meetings’ is shorthand to implementation and delivery team formation and initial discussion of implementation plans.

Implementation took place in three London boroughs (sites 1, 2 and 3) and in Bradford (site 4). Figure 2 depicts the timeline of site engagement and initial actions. Sites 1 and 2 had experience with the previous iteration of UCLP-PRIMROSE (PRIMROSE-A) and began transitioning from PRIMROSE-A to UCLP-PRIMROSE at the start of 2023. Site 4 hosted its first UCLP-PRIMROSE training session near the end of 2022 and site 3 at the start of the QOF year 2023. Sites 3 and 4 began delivering UCLP-PRIMROSE in the new QOF year in 2023. Over the following year, 24 general practices implemented UCLP-PRIMROSE to some extent (14 general practices in site 1, two in site 2, four in site 3 and four in site 4). Seven of these general practices were used as case study practices.

**Figure 3:**
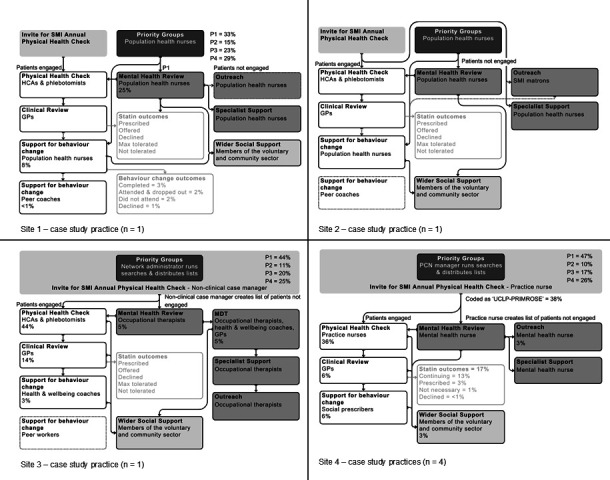
Local adaptation of model delivery. (GP, general practitioner; HCA, health care assistant; MDT, multidisciplinary team; PCN, primray care network; SMI, severe mental illness.) Where arrows have dotted lines, referral pathways were inconsistently used. Unconnected elements of the model represent staff availability but no connection with the model delivery. Percentages of patients accessing each part of the pathway are shown throughout the model for case study sites where available. All figures are percentages of the number of patients on the SMI register at case study practices. Note case study site 4 covers all 4 general practices for this location as data was pulled at primary care network level and not identifiable to specific practices. Light grey boxes indicate additional conversations around of statins and a breakdown of outcomes from behaviour change sessions where they were available.

There was local variation in the way the pathway was adapted, as shown with the case study practices in figure 3. This was in terms of what was delivered (such as only site 1 providing the offer of peer coaching), who was delivering core elements (eg, the mental health review was delivered by population health nurses in sites 1 and 2, occupational therapists in site 3 and a secondary care mental health nurse in site 4) and different referral pathways.

Participants belonged to different organisations, including primary and secondary care. For those who provided demographic characteristics, the mean age was 44 years (ranging from 23 to 66 years), 64% of participants self-defined as holding clinical roles, and the mean time spent in their current role was 14 years (ranging from 2 to 40 years). See table 1 for the breakdown of gender, job role and ethnicity.

**Table 1:** Interview participants’ demographic characteristics

Category	Subcategory	N (%)
Gender	Man	9 (23.1%)
	Woman	21 (53.8%)
	Prefer not to say	1 (2.6%)
	Missing	8 (20.5%)
Ethnicity	Asian or Asian British	4 (10.3%)
	Black, Black British, Caribbean or African	3 (7.7%)
	White	22 (56.4%)
	Other ethnic group	2 (5.1%)
	Prefer not to say	–
	Missing	8 (20.5%)
Clinical role*	Coach (peer or health and wellbeing)	4 (10.3%)
	GP or GP partner	4 (10.3%)
	Nurse (nurse, mental health nurse, population health nurse)	4 (10.3%)
	Pharmacist	1 (2.6%)
	Psychiatrist	2 (5.1%)
Non-clinical role*	Administration or support	3 (7.7%)
	Implementation specialist	3 (7.7%)
	Manager (head of service, lead, primary care network manager, trainee)	5 (12.8%)
	Project or programme manager	2 (5.1%)
	Missing	13 (33.3%)

* Note—two participants held more than one role; therefore, the total per cent for clinical and non-clinical roles is over 100%.

### Integrated findings

We have chosen to present the three main themes from our reflexive thematic analysis, with insight from CFIR, NPT and pathway uptake embedded. We selected to write the results in plain language, rather than using terminology from NPT and CFIR, to increase accessibility. The themes are (1) transformative care - are we looking at it right? (2) developing stable relational and practical foundations within constrained contexts and (3) not business as usual yet: reinforcing, recalibration and re-engagement.

We have labelled quotes with pseudonyms and associated site(s). If participants were connected to the overall implementation of UCLP-PRIMROSE, ‘Across’ has been used. Participant job role and demographic characteristics are not included to maintain anonymity.

#### Transformative care: are we looking at it right?

This theme collates critical reflections on the implementation in the context of NHS systems and culture and expectations related to outputs of clinical improvement for patients. As one participant described, we need to look at things differently when implementing in the *“real world”*:

Avoiding what we've done for generations in the NHS, just believing that all we have to do is articulate the guidance just that bit more clearly … The magic doesn't happen because the real world doesn't allow it to (Oliver, Across).

When implementing UCLP-PRIMROSE, implementation and delivery teams found themselves pushing against a system which was relatively inflexible while also being in transformational flux (the focus at the time being the shift towards ICSs). One participant describes the rigidity of the system, *“We are trying to fit [UCLP-]PRIMROSE into a square box. It’s always going to be problematic for different technical reasons”* (Lily, Site 1). Therefore, participants commonly spoke of UCLP-PRIMROSE as a first test for the implementation of integrated working and approaches. This quote relates to care being duplicated across primary and secondary care, while UCLP-PRIMROSE aimed to integrate care across settings:

I agree that that’s one of the selling points of [UCLP-]PRIMROSE, the knitting together [of different care being delivered for patients with SMI], and in fact that these [physical health] checks are happening in two different places [primary and secondary care] without regard to each other (Robert, site 4).

Key ongoing barriers to this were primary and secondary care electronic patient record systems not being connected and a lack of data sharing agreements between the different settings. Often, implementation teams felt they had limited power, knowledge and resources to easily solve such problems, which were deemed to be *“a symptom of the wider system”* (Poppy, sites 1 and 2).

Findings from qualitative analysis taken with pathway uptake data highlighted that elements needing less adaptation (more compatible with existing work) and were interpreted as less complex were more consistently delivered and from earlier on. Broadly across the 24 general practices, there was consistent delivery of health checks, clinical reviews and wider support (the UCLP-PRIMROSE model specifies wider support as using existing structures to address additional patient priorities, such as financial concerns).

The consistently delivered health check was frequently connected to centrally mandated incentivised targets (QOF screening targets in SMI). This was a priority across general practices, but many participants discussed that focusing on screening alone was not complete care or supportive of efforts with UCLP-PRIMROSE implementation. Kath outlined her perspectives on the targets:

What’s important for patient care. How are we gonna save the most lives? … NHS England haven't learnt that. NHS England are still setting us targets of ‘measure the sugar’, no, get the sugar low (Kath, site 3).

Across the sites involved in UCLP-PRIMROSE, we witnessed the knock-on impact of how much resource could be dedicated to work which was not connected to the health check, especially at certain times of the financial year. For example, efforts in the last quarter were dedicated to trying to reach QOF screening targets, drawing effort away from other elements of care in UCLP-PRIMROSE.

Consequently, participants described that a shift in culture was needed in terms of the type of care that was being delivered via UCLP-PRIMROSE (holistic and patient-centred with a focus on lifestyle change to reduce CVD risk) and that more was needed to support the readiness of staff. One participant specifically draws on the change needed in terms of outcomes, *“You have to value the long, slow hard grind over the fast hits of large numbers”* (Lucas, sites 1 and 2).

This required shift in expectations for quick clinical outcomes was extended by participants to research timelines and strategy cycles too, which seemed unrealistic:

The problem with the data and with the study is that change for people is quite slow and we probably won't even know if our interventions have made a difference for many years (Olivia, site 4).

However, our research timeline allowed us to capture the first year of implementation iteration and fluctuation, particularly of certain pathway elements. There was less consistent implementation of the search stratification tool and related actions (19 practices), the full scope of the non-engagement element, including outreach home visits (six practices), and provision of care from peer coaches (one practice). In addition to compatibility with policy and culture, this was connected to compatibility with existing successful ways of working, staff availability, turnover and capacity and how complex the element was to set up and execute. As an example of complexity, the non-engagement element was best supported by skilled staff, dedicated time for admin, digital connectivity between primary and secondary care and the need for consideration of risk assessments, resources for staff travel and out of hours access to fridges to store blood samples.

Moreover, establishing referral pathways and integrated working (discussed further in the next theme) was hindered by poor understanding of staff role definitions, their boundaries and their importance (felt most strongly with health and wellbeing coaches, peer coaches and occupational therapists). The low uptake of peer coaching was surprising and concerning to a few participants, as described in this quote:

I regard peer support as one of the really useful mechanisms for making this framework effective for the patient, not for the clinic, for the patient. And I wouldn't have expected in 2023, almost 2024, for it to be so difficult to get peer coaches involved (May, Across).

#### Developing stable relational and practical foundations within constrained contexts

This theme unpacks the complexity of taking an evidence-based framework to practice, manoeuvring a context with stretched resources (organisational complexity), while building relationships and navigating political and hierarchical challenges (value and sociocultural complexity). One participant highlighted this succinctly when describing the leap between theory and practice:.

It’s [a] good project. I think the theory behind it is wonderful. It’s just that we need people to sort of back it up (Gary, site 2).

The research team reflected that the initial uptake was less influenced than expected by the design of UCLP-PRIMROSE and its materials or training attendance (although when delivered, training did support understanding of UCLP-PRIMROSE and the development of networks of participation). For the latter, UCLP-PRIMROSE training was not delivered in sites 1 and 2, key champions were unable to attend training in site 3, and across sites some individuals who attended training did not go on to deliver UCLP-PRIMROSE. Rather, the variation in practice and staff readiness and availability of resources meant skilful, and dedicated implementation leads, project managers and influential champions were key to evolving initial interest into concrete actions. One participant described a lack of GP engagement as a showstopper for site 2:

Without GP engagement, you're not going to do anything. No, unquestionably … [A] GP who is enthusiastic for it to happen in a practice is the single most important thing (Lucas, sites 1 and 2).

Consistent involvement of leadership and/or influential staff representatives, such as a clinical director in site 1, and individuals with existing cross-cutting roles, such as GP mental health leads in sites 3 and 4, were seen as particularly important. They could support with bridging across hierarchical structures, building connections across teams and advocating for resource release. When leaders were not engaged or there were internal political struggles between leaders, this was identified as a barrier. For example, in sites 1 and 2, a new post of ‘population health nurse’ was previously created to change the language and expectations over roles and responsibilities in relation to SMI physical health and ring-fence resource. However, disconnected senior and middle management priorities led to conflicting expectations and less support for relationship building between staff in these delivery roles.

Champions were also important to motivate the system and to provide local coordination of allocated resources, but the efforts of champions need to be combined with the strategic priorities of providers (secondary and primary) and for staff to have allocated time in their job plans for UCLP-PRIMROSE activity. Additionally, the limitation in the reach of individual champions was highlighted in meetings in site 4 after a series of practice visits by a researcher and project manager. They noted that understanding and engagement tended to get weaker in practices the further away from the key champion, in terms of social network and their physical location. This was also found in follow-up interviews for sites 1, 2 and 3, where participants described that the space for collective discussion and ongoing reflection was narrowed to people in the core implementation group who had limited ripples of influence and power. Therefore, reliance solely on champions may create unstable ground for uptake and pockets of best practice. Early and ongoing collaboration, which included leadership, driven by a project manager and a central implementation team, was important to support implementation foundations.

However, getting representation of staff together from across teams was described as onerous. Implementation required upfront commitment of resources, both practical (time to attend meetings, model mapping and training, but also resources such as rooms, equipment and electronic patient record availability) and psychological (staff having the cognitive capacity to consider something new). An example of psychological resources being facilitated was evident in site 3, with leaders encouraging cross-team working and dedicating time for projects. This was seen as part of normal expectations for staff and time allocated to training to support staff readiness. However, in sites 1 and 2, while there were ongoing positive discussions such as ring-fencing time for training, these discussions were commonly not translated into overt action through the release of staff time. When considering enabling cultures to embed new learning, it was important that staff felt valued, that their opinions were sought and that adequate support was provided to assist their efforts in trying something new.

#### Not business as usual yet: reinforcing, recalibration and re-engagement

This theme captures the need to revisit the above implementation foundations, as well as accountability in actions and reporting, and consistency of pathway delivery. For all sites, progress was made with implementation efforts of UCLP-PRIMROSE across the relatively short timeframe, but while there was a desire for the intervention to become business as usual, this was not achieved during the study period.

During the follow-up interviews and in meetings near the end of the research, there was discussion about progress and impact from UCLP-PRIMROSE and what this meant for implementation efforts for the new financial year. There was still support for UCLP-PRIMROSE overall and for most of its components. There was agreement in all sites that it was a way to meet the needs of their patients with SMI, reduce health inequalities, and overall was how care should be delivered. For the latter point, one participant summarised, *“It feels less like a tick box and more like patient care”* (Stephen, site 3).

This was further reinforced through personal experience or information shared through qualitative feedback. For participants delivering the support for the behaviour change element of UCLP-PRIMROSE, staff mentioned the positive impact of building a therapeutic relationship for *“small wins”* (Dave, site 1) and the transformational outcomes:

He was quite an unwell person. And to see you where he is now and then to see him on this programme …. Yeah, it meant a lot. I found it heartwarming really (Ellen, site 1).

Participants in site 1 were able to give the strongest feedback related to positive changes. They had previous experience of PRIMROSE-A and therefore had greater confidence with pathway elements and reported positive unforeseen consequences. For example, using their skills across patients, not just those within UCLP-PRIMROSE, as *“It sort of comes natural when you are approaching the patient, you are able to sort of approach the person from the holistic [viewpoint]”* (Joanna, site 1).

However, there was less quantitative feedback on pathway uptake or clinical changes to support sustainability. Site 3 was unique in its ongoing efforts to use the availability of quantitative data related to QOF as a proxy for progress and to push engagement, momentum and spread of UCLP-PRIMROSE (leaning into competitiveness in general practice and prioritising progress with incentivised targets). For sites 1, 2 and 4, methods for recording UCLP-PRIMROSE care were inconsistent, and difficulties with automatic data extraction hindered reporting. Some participants reported frustration with UCLP-PRIMROSE templates used on electronic patient records to log consultations. Other participants talked about the administrative requirements to support delivery and data collection, noting these had not been fully considered, plus insufficient staff with skills or time to address emerging issues.

Across sites, participants discussed the need to improve data recording and reporting to enable oversight and ongoing evaluation of outcomes resulting from UCLP-PRIMROSE. This included oversight to make sure the quality of the intervention is maintained. For example, at site 4, the absence of automatic reporting for new statin prescriptions after a UCLP-PRIMROSE health check led to manual record searching. These searches found that a percentage of the 'target’ population prescribed a statin rose by 20% when comparing 2022/2023 (no UCLP-PRIMROSE) to 2023/2024 (provision of UCLP-PRIMROSE). In site 1, the team has been able to spread to many practices, but some reflected that they had limited oversight of delivery completeness and its outcomes. This was connected to potentially risking quality and future sustainability:

I wonder whether we've expanded too quickly 'cause it’s an achievement … When we spread but nothing happens, we kind of dilute the substance of the intended purpose (Benjamin, sites 1 and 2).

However, ongoing evaluation discussions helped teams to learn about the value of core UCLP-PRIMROSE elements and consider which should be taken forward unchanged, require modification, or may be deimplemented. Site 4 was in conversation about trying a different approach to the non-engagement part of the pathway, agreeing on its benefit, while considering deprioritising actions around the search stratification tool. For the latter, good practices around recalling for health checks had already been in place, and use of the stratification tool had resulted in substantial administrative burden:

The whole stratification bit, they complained, the staff members … it wasn't very straightforward, it was a bit complex (Reese, site 4).

Staff in sites 1 and 2 discussed how a large amount of time and resources had been dedicated to overcoming the barrier of enabling peer coaches to access and record on primary care electronic patient records to deliver UCLP-PRIMROSE. As they were unsuccessful in trying a flexible approach in different practices, but saw this element as integral, one participant suggested they now needed to come up with an approved solution for patient record access and make it mandatory. Site 3 was also increasing efforts to implement the peer coaching support for UCLP-PRIMROSE, in this case agreeing to dedicate resource to recruitment, and was revisiting staff capacity within the non-engagement part of the pathway.

These discussions were commonly framed within wider considerations around building capacity into the NHS through supportive structures to be able to spread innovations like UCLP-PRIMROSE. As mentioned in the previous themes, cognitive capacity is needed to consider uptake and local adaptation and then facilitate within-system learning, problem solving and iterative implementation. This raised additional questions around health inequalities, especially if innovations are only taken up by teams who are already performing well:

The practices who are really struggling just with the day job and can't offer anybody appointments struggle most with people with complex problems like severe mental illness, and they're much less likely to take [it] on (Oliver, Across)*.*

Those in implementation roles, rather than delivery roles, held realistic expectations around sustaining and spread. They accepted that implementation slowed down at times, and there was a need for recalibration, for example, with their approach to reporting and recording and re-engagement of staff to deliver core components of the pathway. They also stressed that embedding UCLP-PRIMROSE for sustainable delivery needs the oversight and involvement from the right people with skills, time and passion, *“as it has proven to be something more of a fragile flower”* (Lucas, sites 1 and 2).

## Discussion

We evaluated the implementation of UCLP-PRIMROSE across three London boroughs and in Bradford, gaining insights about what worked well and what did not, the barriers and enablers to implementation, and the underpinning processes. This spanned a unique time in the NHS landscape, including the aftermath of the COVID-19 pandemic,^37^,^39^ meaning there was uncertainty and unrest.^40 41^ However, there was a major policy emphasis on SMI^6^,^8^ and the formation of new ICSs in 2022,^14^ which should have been supportive for innovations such as UCLP-PRIMROSE.

UCLP-PRIMROSE matched NHS policy, addressing most of the 10 key actions needed to improve the physical health of people living with SMI^42^ and has since been recommended in best practice national guidance.^43^ There was an appetite for UCLP-PRIMROSE, and pockets of best practice emerged across the four localities. Nonetheless, we observed challenges to delivering the full pathway. Implementation teams worked iteratively, finding pathway elements most compatible with existing ways of working quickest to set up, and committing to further resourcing elements with perceived value (non-engagement and peer support elements). Teams also learnt which components did not add value or required modification, and we observed discussions around potential deimplementation of pathway elements in one site (namely the search stratification tool). This aligns with the foundational work on NPT, which explains how interventions are embedded within organisations, with a focus on the importance of iterative feedback and re-engagement for successful long-term adoption^31^.

Our study highlighted the need for skilled champions and human and technical resource but importantly also required supportive cultures to support navigating contextual, structural and project management misalignments. Translation of UCLP-PRIMROSE into practice needs upfront resources, specifically in staff having adequate time and cognitive capacity to consider UCLP-PRIMROSE as important compared with other responsibilities, a team to support project management to operationalise UCLP-PRIMROSE, and administrative support. However, also crucial was a supportive learning culture, which encourages accountability, consistency and reporting (eg, use of key performance indicators) around their care delivery, with space for adequate reflection, problem solving and development of trusting relationships. Other studies have shown the value of reflective practices and knowledge-sharing environments to enhance learning within the NHS, emphasising how structured time for reflection and iterative learning supports the advancement of healthcare practices^44^. Creating space for reflection helps healthcare teams to adapt and continuously improve, supporting a culture of within-system learning or quality improvement.^45^ This will be particularly important, as care teams increasingly work in a collaborative way across care boundaries.

When conducting this work, ICSs were in their infancy,^14^ which meant teams were navigating systems with promised, rather than achieved, systems integration. System-level reforms in healthcare can strain interprofessional collaboration, especially when existing roles are reshaped to accommodate new initiatives, impacting overall effectiveness and creating internal challenges for implementation.^46^ Practically, teams faced challenges with implementation when working across care boundaries, developing a local understanding of job roles and navigating capacity. This led to bigger questions, often out of the scope or reach of implementation teams around data sharing agreements, technological compatibility for recording data and less successful referral pathways developed into less familiar roles. These findings, particularly continued issues with interoperability, are already known. For example, Greenhalgh and Papoutsi’s^47^ work delves into the complexities of implementing cross-sector care, highlighting barriers such as data-sharing issues, technological compatibility and interorganisational challenges, and provides a foundation for understanding the interoperability issues encountered with UCLP-PRIMROSE.

Moreover, compounding efforts in terms of the wider context were competing messages related to QOF targets. Health checks benefit care through continued emphasis on physical health in patients with SMI, providing a base framework for patient recall, screening and recommended support for patients when needed. The QOF’s incentivisation approach has encouraged standardisation of specific health check screening, but it has also had the unintended consequence of disincentivising other forms of non-targeted preventive care, potentially reducing uptake of holistic, evidence-based interventions.^48^ Target thresholds for patients receiving all six elements of the physical health check under the QOF were set to 60%–75% of patients having the full check for 2024–2025^49^, but in NHS England planning guidance, targets for SMI physical health checks were absent.^50^ Our work supports previous research suggesting the guidelines are not followed and completed in their entirety,^51 52^ underpinning the ongoing importance of The Lester tool: ‘Don’t Just Screen, Intervene’.^53^ However, removal of incentivisation may risk a reduction in health checks as well as relevant follow-up care and would require champions in the system to advocate for continuing and expanding the activity.

### Strengths and limitations

A core strength of this work was its interdisciplinary team, who brought different perspectives in terms of clinical, implementation and research experience. This facilitated the collection of in-depth data and the use of complementary approaches to analysis.

We drew on the accessibility of reporting from reflexive thematic analysis in this paper, the content of each theme strengthened by the integration of CFIR, NPT and insights from uptake data. The aim of this paper was to report on implementation, and our methodological engagement with CFIR and NPT will be the topic of further papers. However, while the qualitative analysis methods worked cohesively and could be triangulated with our quantitative findings, there were some limitations in our work. This included participant recruitment and gaps in the availability of quantitative data.

For patient recruitment, patients preferred to provide informal patient feedback rather than to take part in interviews. Feedback from staff and patients was that the recruitment process was too lengthy. Staff let us know that they did not have time for the recruitment procedures or had limited awareness of what the research involved for patients. For patients, the length of time before they could speak to a researcher meant they were no longer interested, or that their care related to UCLP-PRIMROSE did not stand out from their other care.

Considering our quantitative data, each site used a different method of recording, locally adapting this as part of implementation. This was a strength in our work in that it provided us with a window into implementation outside the constraints of more structured research, such as clinical trials. However, this led to challenges with inconsistent recording practices and assigning clinical actions to UCLP-PRIMROSE care (such as statin prescription or invitation to a consultation).

### Conclusions

This study examined the implementation of UCLP-PRIMROSE across Yorkshire and London during a period of substantial transition, revealing its alignment with policies aimed at improving the physical health of individuals with SMI and the direction of travel for integrated care. There are important considerations required to inform wider and sustainable roll-out in the NHS and advance the care for patients with SMI. While uptake was promising in some areas, challenges emerged around consistent pathway delivery due to structural complexities, local resource constraints and interoperability issues.

## Supplementary material

10.1136/bmjopen-2025-105511 online supplemental file 1

10.1136/bmjopen-2025-105511 online supplemental file 2

10.1136/bmjopen-2025-105511 online supplemental file 3

10.1136/bmjopen-2025-105511 online supplemental file 4

10.1136/bmjopen-2025-105511 online supplemental file 5

## Data Availability

Data are available upon reasonable request.
