# Supplementary material for: Bridging the gap in the UK’s National Health Service integrated care systems: insights from a mixed methods implementation evaluation of UCLP-PRIMROSE, a care innovation to reduce physical health inequalities for people with severe mental illness
Source: BMJ Open. 2026 Jan 27;16(1):e105511. doi: 10.1136/bmjopen-2025-105511 (PMC12853453; doi:10.1136/bmjopen-2025-105511)
Supplement: online supplemental file 1 [file bmjopen-16-1-s001.pdf]

TIDieR

UCLPartners-PRIMROSE

Why:

The PRIMROSE programme of research developed a 6-month nurse-led primary care service for managing cardiovascular disease (CVD) risk in people with severe mental illness (SMI). This involved eight 30-minute appointments focused on behaviour change strategies and goal setting to reduce CVD risk factors. PRIMROSE was not clinically superior to treatment as usual in a cluster trial on the primary outcome measure but was positive on secondary outcomes including fewer adverse events, lower total health care costs, and reduced psychiatric inpatient admissions. Based on the trial and wider programme results, PRIMROSE was adapted to include better prioritisation of CVD medications and provide additional social support via peer coaching (becoming PRIMROSE-A), and incorporate latest CVD evidence (becoming UCLPartners-PRIMROSE).

UCLPartners-PRIMROSE or UCLP-PRIMROSE is the integration of PRIMROSE-A with the UCLPartners Proactive Care Frameworks (see here for the evaluation). It was developed to maximise the benefits of the SMI physical health check and to help people with SMI to receive the best care for their physical health as well as their mental health. UCLP-PRIMROSE was also designed to build capacity within healthcare delivery by utilising the wider workforce and integrating care across boundaries of primary, secondary and voluntary care.

UCLP-PRIMROSE includes five core components:

1. Search stratification - patients with SMI are stratified into four priority cohorts based on their level of CVD risk, completed using a search and risk stratification tool (referred to in the rest of the paper as the search stratification tool) imported into electronic patient record systems. This informs the order patients will be invited for their SMI annual health check.
2. Physical health check – those who engage with the invites will receive a holistically delivered health check.
3. Clinical review – a consultation to discuss the results from the health check. An appropriate care plan would be developed informed by the patient’s needs and preferences. This could include medication optimisation, support for behaviour change and peer coaching, and wider social support (signposting to address additional needs such as housing).
4. Delivery of the care plan.
5. Non-engagement support - for patients who do not respond to the health check invite, guidance to increase engagement has been built with a mental health desktop review coupled with outreach and specialist support if needed.

What (material):

The publicly available package for UCLP-PRIMROSE is accessible from the UCLPartners website. This includes an implementation guide, NHS England recommended training lesson plans, videos and eLearning (also available on the NHS Learning Hub), and manuals for each of the core components.

What (procedures):

The activities related to UCLP-PRIMROSE are presented in the UCLP-PRIMROSE pathway:

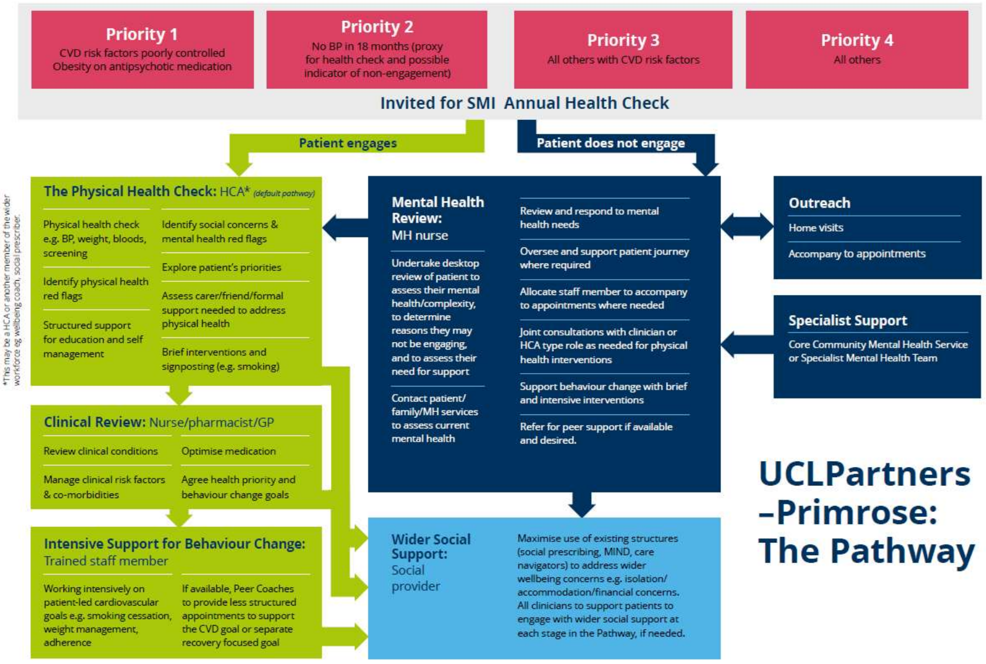

Who provided:

Some examples of staff who may be appropriate to provide each step are provided on the UCLP-PRIMROSE pathway, however who provides each component of UCLP-PRIMROSE is adapted to local resource and ways of working. Staff members may be from primary, secondary, or voluntary care. A implementation map has been created to support local teams discuss and plan who will deliver what, how pathways between care elements will work, and considerations of how teams would like to measure delivery and outcomes:

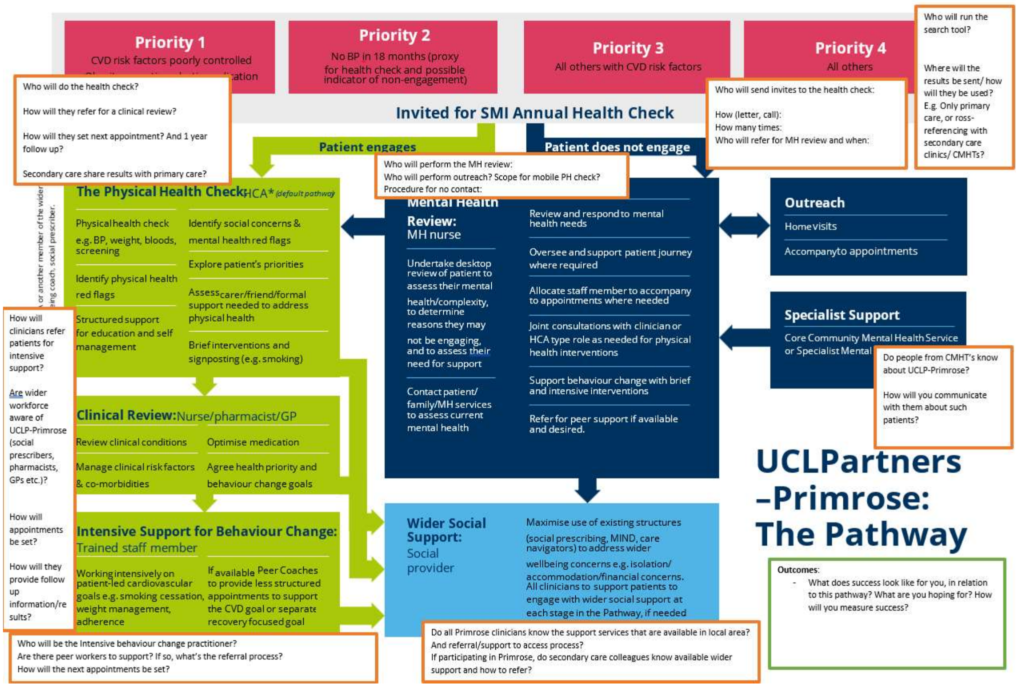

All staff members supporting or delivering UCLP-PRIMROSE are encouraged to participate in UCLP-PRIMROSE training (this extends to staff working in the GP practice who may not have direct roles such as receptionists). The video resources were created to be engaged with before in-person training, with staff of all expertise / backgrounds identifying gaps in their knowledge and engaging with the relevant resource (for example staff with no mental health training would engage with videos introducing SMI). In person training supports with knowledge about UCLP-PRIMROSE and its delivery and specific components including role play around behaviour change conversations. It is recommended regular training sessions are offered to re-fresh knowledge and skills, as well as to support any new staff.

How (mode of delivery; individual or group):

UCLP-PRIMROSE delivery is tailored to individual patient needs. Staff use the pathway as a guide, balancing best practice recommendations with patient priorities, as well as their level of engagement.
For example if a patient is engaged with their care and therefore attends a GP practice for a face to face health check and clinical review, any conditions may be well managed and no risks flagged clinically and no concerns raised by the patient. This may therefore be the extent of this patients involvement with the UCLP-PRIMROSE pathway.
However, if a patient engages and risks or patient concerns are identified, relevant support would be agreed and actioned. This could result in a variety of support which might include the support for behaviour change to reduce CVD risk, eight 30-minute appointments which mainly are delivered face to face, but with the option of telephone appointments if needed.
Alternatively, for patients not engaging with their care additional modes of delivery might be employed such as outreach via home visits.
Please see the manuals for further details on how UCLP-PRIMROSE is delivered.

Where:

In our research to explore the implementation of UCLP-PRIMROSE there was uptake across Yorkshire and London. The primary location of care delivery was primary care, in GP practices. However, support from secondary care and voluntary care was integrated.

When and how much:

Guidance for the delivery of UCLP-PRIMROSE at each stage is detailed in the manuals created for each core component of the model. For example for the 'Support for Behaviour Change Component' provides a suggested schedule for the eight appointments including duration (varies from 20 to 40 minutes), when to deliver, and what to include in each. Similar details are specified for the suggested four appointments of 'Peer Support'.
However, duration, number of sessions, and their schedule should be tailored to patient need.

Tailoring:

Yes, as specified in previous sections, whilst UCLP-PRIMROSE has a comprehensive package of training and guidance, the intervention delivered should be tailored to local resource, ways of working and individual patient need.

How well (planned):

In our implementation research we designed a mixed-methods observational study, and aimed to explore variation in delivery. This was not a clinical trial with planned and specified implementation.
