# Supplementary material for: Bridging the gap in the UK’s National Health Service integrated care systems: insights from a mixed methods implementation evaluation of UCLP-PRIMROSE, a care innovation to reduce physical health inequalities for people with severe mental illness
Source: BMJ Open. 2026 Jan 27;16(1):e105511. doi: 10.1136/bmjopen-2025-105511 (PMC12853453; doi:10.1136/bmjopen-2025-105511)
Supplement: online supplemental file 2 [file bmjopen-16-1-s002.doc]

**Standards for Reporting Qualitative Research (SRQR)**

O’Brien B.C., Harris, I.B., Beckman, T.J., Reed, D.A., & Cook, D.A. (2014). Standards for reporting qualitative research: a synthesis of recommendations. *Academic Medicine, 89(9)*, 1245-1251.

| **No. Topic** | **Item** |  |
| --- | --- | --- |
| **Title and abstract** |  |  |
| S1 Title | Concise description of the nature and topic of the study identifying the study as qualitative or indicating the approach (e.g., ethnography, grounded theory) or data collection methods (e.g., interview, focus group) is recommended | Page 1: Nature of the topic has been included – implementation evaluation / intervention integrating mental and physical health. We have included ‘mixed methods’ in our title. We have selected this rather than listing all the data collection and analysis methods as that would make the title too lengthy. |
| S2 Abstract | Summary of key elements of the study using the abstract format of the intended publication; typically includes objective, methods, results, and conclusions | This has been included on pages 2, 3, and 4. |
| **Introduction** |  |  |
| S3 Problem formulation | Description and significance of the problem/phenomenon studied; review of relevant theory and empirical work; problem statement | This background of and significance of the topic (understanding barriers, facilitators and process of implementation of evidence-based care for people with severe mental illness at risk of cardiovascular disease) is across pages 6 to 9. |
| S4 Purpose or research question | Purpose of the study and specific objectives or questions | The aims of the current research are outlined on page 9. |
| **Methods** |  |  |
| S5 Qualitative approach and research paradigm | Qualitative approach (e.g., ethnography, grounded theory, case study, phenomenology, narrative research) and guiding theory if appropriate; identifying the research paradigm (e.g., positivist, constructivist/interpretivist) is also recommended | The methods used in this research have been documented across pages 9 to 16.  In our original submission we tried to be comprehensive in our presentation of approaches but acknowledged not all information could be included due to the word limit of the journal. In response to reviewer comments the methods section has been substantially expanded, allowing us to provide additional description and rationales. |
| S6 Researcher characteristics and reflexivity | Researchers’ characteristics that may influence the research, including personal attributes, qualifications/experience, relationship with participants, assumptions, or presuppositions; potential or actual interaction between researchers’ characteristics and the research questions, approach, methods, results, or transferability | Additional information about the authors who collected data and performed the initial analysis has been included on page 37 to aid in interpretation. |
| S7 Context | Setting/site and salient contextual factors; rationale^a^ | On page 16 and 17 we have included a description and a diagram (included sites and onboarding timelines). |
| S8 Sampling strategy | How and why research participants, documents, or events were selected; criteria for deciding when no further sampling was necessary (e.g., sampling saturation); rationale^a^ | Our sampling strategy was different across the methods of data collection. Where relevant we have provided more detail on the sampling – such as selection of case study sites on page 12 and that involved staff were emailed to be invited to take part in interviews (pages 10). We have also provided a section titled ‘sample size’ which discusses our rationale, on page 12. |
| S9 Ethical issues pertaining to human subjects | Documentation of approval by an appropriate ethics review board and participant consent, or explanation for lack thereof; other confidentiality and data security issues | We have specified we received a favourable opinion from NHS Research Ethics Committee (reference: 20/WS/0153; IRAS: 285554) on page 9. |
| S10 Data collection methods | Types of data collected; details of data collection procedures including (as appropriate) start and stop dates of data collection and analysis, iterative process, triangulation of sources/methods, and modification of procedures in response to evolving study findings; rationale^a^ | The data collection section in this paper starts on page 10 – we documented the different ways we collected data such as interviews and collecting process data documents, as well as the overall timeframe of the study (March 2022 – August 2024) and relevant timeframes within in this, such as 9 months between interviews (rationale for this is provided on page 10). |
| S11 Data collection instruments and technologies | Description of instruments (e.g., interview guides, questionnaires) and devices (e.g., audio recorders) used for data collection; if/how the instrument(s) changed over the course of the study | We have included our interview topic guides in the additional materials of the paper for further information and details of the recording (see page 11). |
| S12 Units of study | Number and relevant characteristics of participants, documents, or events included in the study; level of participation (could be reported in results) | We have revised our presentation of units of study in response to our reviewers’ comments. Therefore, some information, such as number of interview participants sits within the methods (pages 9-13), and the sites included and participant demographics are reported at the start of our results across pages 16, 17, and 18. |
| S13 Data processing | Methods for processing data prior to and during analysis, including transcription, data entry, data management and security, verification of data integrity, data coding, and anonymization/deidentification of excerpts | How interview data was recorded and transcribed, and who by is reported on page 11. The transcripts were de-identified, but as specified on page 11 we also elected not to include participant demographic characteristics or job roles with the quotes in our write-up to maintain anonymity. |
| S14 Data analysis | Process by which inferences, themes, etc., were identified and developed, including researchers involved in data analysis; usually references a specific paradigm or approach; rationale^a^ | Our reporting of data analysis starts on page 13 covering reflexive thematic analysis and CFIR for our qualitative work, and approach to quantitative on page 15. The integration was also key to document, including who completed this and what the process was, which is also on page 15 (the rationale for use of different approaches is on page 13). |
| S15 Techniques to enhance trustworthiness | Techniques to enhance trustworthiness and credibility of data analysis (e.g., member checking, audit trail, triangulation); rationale^a^ | Throughout the work checks were in place to enhance trustworthiness, with references where relevant such as comparison of coding between researchers, audit trails created with our work with CFIR described on page 14 (NVivo, memos, and then a matrix) and our integration on page 15, and collaboration with our interdisciplinary team (page 15) in our analysis and interpretation of findings. |
| **Results/Findings** |  |  |
| S16 Synthesis and interpretation | Main findings (e.g., interpretations, inferences, and themes); might include development of a theory or model, or integration with prior research or theory | Reporting of our integrated findings is across pages 16 to 29. This is the presentation of three themes from the integration of our analysis approaches and subsequent application of NPT. |
| S17 Links to empirical data | Evidence (e.g., quotes, field notes, text excerpts, photographs) to substantiate analytic findings | We have supported our findings section (pages 16-29) with quotes and reference to uptake data where relevant. |
| **Discussion** |  |  |
| S18 Integration with prior work, implications, transferability, and contribution(s) to the field | Short summary of main findings; explanation of how findings and conclusions connect to, support, elaborate on, or challenge conclusions of earlier scholarship; discussion of scope of application/generalizability; identification of unique contribution(s) to scholarship in a discipline or field | Our discussion spans pages 29 to 33 where we have summarised and elaborated on our findings, connecting these to relevant contextual information and literature. |
| S19 Limitations | Trustworthiness and limitations of findings | We have included a strengths and limitations section on page 33. |
| **Other** |  |  |
| S20 Conflicts of interest | Potential sources of influence or perceived influence on study conduct and conclusions; how these were managed | Competing interests have been documented on page 5 and 6. |
| S21 Funding | Sources of funding and other support; role of funders in data collection, interpretation, and reporting | Our funders are reported on page 5. |

^a^The rationale should briefly discuss the justification for choosing that theory, approach, method, or technique rather than other options available, the assumptions and limitations implicit in those choices, and how those choices influence study conclusions and transferability. As appropriate, the rationale for several items might be discussed together.
