# Supplementary material for: Bridging the gap in the UK’s National Health Service integrated care systems: insights from a mixed methods implementation evaluation of UCLP-PRIMROSE, a care innovation to reduce physical health inequalities for people with severe mental illness
Source: BMJ Open. 2026 Jan 27;16(1):e105511. doi: 10.1136/bmjopen-2025-105511 (PMC12853453; doi:10.1136/bmjopen-2025-105511)
Supplement: online supplemental file 3 [file bmjopen-16-1-s003.docx]

# UCLP-PRIMROSE study – Qualitative Interview Topic Guide

# Health care professional experiences

1. What is your professional background and your current job role?
2. How did you become involved in delivering the Primrose intervention?
3. What was your understanding of Primrose at the start?
   1. What were your motives for delivering Primrose?
   2. Did you have any concerns?
4. What was the approach for implementing Primrose?
   1. How did you feel about your skills to deliver the intervention?
   2. What did you think of the training / resources?
5. What was your role specific to delivering the Primrose intervention?
6. What are your personal experiences of delivering Primrose since its introduction?
7. How far have you (and/or others) achieved what you set out to do?
8. What have been the challenges to implementation?
   1. Were you able to overcome some or all of these barriers? Could you provide an example of overcoming a barrier?
9. What helped with the implementation of Primrose?
   1. What was the support like in terms of implementation?
   2. How have you found implementing Primrose with patients?
10. What aspects of the intervention do you think worked well and which didn’t work well?
11. Do you have any next steps in place for the PRIMROSE intervention?
12. What have you learned during this process?
    1. Reflecting on your experience, what do you think the benefits/advantages might be of the intervention?
    2. What do you think might be the disadvantages?
    3. If you were implementing Primrose again, what would you do differently?
13. What are your thoughts on providing this service long term?
14. Do you think PRIMROSE may be a suitable intervention for other practices/GPs across the country to implement? Why or why not?

## Conclude discussion

I just want to finish by asking you:

1. Is there anything that has not been discussed that you would like to raise?
2. Are there any other comments/questions or ideas?
