# Supplementary material for: Bridging the gap in the UK’s National Health Service integrated care systems: insights from a mixed methods implementation evaluation of UCLP-PRIMROSE, a care innovation to reduce physical health inequalities for people with severe mental illness
Source: BMJ Open. 2026 Jan 27;16(1):e105511. doi: 10.1136/bmjopen-2025-105511 (PMC12853453; doi:10.1136/bmjopen-2025-105511)
Supplement: online supplemental file 4 [file bmjopen-16-1-s004.docx]

# UCLP-PRIMROSE Study – Qualitative Interview Topic Guide

# Service user experiences

How long have you received support for your mental health?

## Experience of using the intervention

- Thinking right back to when you were first approached about improving your physical health, how were you approached about this?
  - By whom?
  - How were the sessions arranged?
  - Any difficulties?
  - Anything that helped make accessing the intervention easier?
  - Was the support something you felt like you needed?
- How many appointments did you have?
  - If you missed any, is there anything that might have helped you to continue with the sessions?
  - If you attended all, what was it that drove you to keep attending?
- Tell me about your care…
  - Did you review your medications?
  - Did anyone involve a friend or carer and how did you feel about that?
  - Did you receive any calls or a home visit to encourage you to attend your appointments?
  - Did anyone offer you any support for your housing or your finances?
  - Did you change any of your medicines?
- Can you tell me about your initial goals *insert goal*, what did you hope to achieve with this support?
  - E.g. setting goals about diet, exercise, losing weight, advice on cutting down/stopping smoking, support services available, information on medication (statins/antihypertensives), reducing or stopping drinking alcohol etc.
- How have conversations with your General Practitioner/Healthcare Practitioner/nurse/peer coach gone when looking at how to achieve those goals?
  - Do you feel you achieved your goal?
  - How did you find the care you received?
  - How did it go?
  - Was the support communicated clearly to you?
  - Were the appointments useful?
  - Did anything influence your decision to attend the appointments? (e.g., were you supported to them?)
  - We are interested in exploring equality for people accessing services and how this new support provided for physical health e.g. goal setting with a healthcare professional may be experienced by people differently, therefore was there anything that made you feel more or less supported related to your personal characteristics? [such as your ethnicity, age, gender, sexuality]
- Did you experience any difficulties with your care? Were there any problems or concerns?
  - If so, what? How could these have been managed?
  - If no, was there anything that made it easier?
- Overall, how satisfied were you with your care and the process in which you received it?
  - Any further comments, e.g., could anything be improved?
  - Would you recommend this intervention to someone else? Why/why not?

## Conclude discussion

I just want to finish by asking you:

- Is there anything that we have not talked about that you would like to raise in regards to your care?
- Are there any other comments/questions or ideas?
