# Supplementary material for: Bridging the gap in the UK’s National Health Service integrated care systems: insights from a mixed methods implementation evaluation of UCLP-PRIMROSE, a care innovation to reduce physical health inequalities for people with severe mental illness
Source: BMJ Open. 2026 Jan 27;16(1):e105511. doi: 10.1136/bmjopen-2025-105511 (PMC12853453; doi:10.1136/bmjopen-2025-105511)
Supplement: online supplemental file 5 [file bmjopen-16-1-s005.docx]

Table of NPT constructs mapped to reflexive thematic analysis codes and CFIR constructs

| **NPT Constructs** | **Reflexive thematic analysis codes** | **CFIR Constructs** |
| --- | --- | --- |
| Implementation contexts | COVID-19, Staff turnover, Awareness, Flexibility | Tension for change, Compatibility, Available resources, Planning, Adaptability |
| Adaptive execution | Context, Support, Training Coordination, Engagement and outreach | External policy and incentives, Compatibility, Available resources, Structural characteristics |
| Negotiating capacity | Competing priorities, Capacity, Staff time | Relative priority, Available resources, Compatibility, Opinion leaders, Leadership engagement, Structural characteristics |
| Reframing organisational logics | Practice characteristics, Existing structures, Structural challenges | Compatibility, Learning climate, Leadership engagement, Networks and communication |
| Coherence building | Adapting locally, Operationalization, Complexity, Collaborative development, Leadership, Training, Structural challenges | Evidence strength and quality, Relative advantage, Patient needs and resources, Cosmopolitanism, Access to knowledge and information, Networks and communication, External change agents, Planning, Complexity, Compatibility, Culture, Tension for change, Relative priority, Intervention source |
| Cognitive participation | Champions, Engaging, Knowledge sharing, Scepticism, Clinical GP engagement, Personalised service, Perceived need | Networks and communication, Other personal attributes, Opinion leaders, Formally appointed internal implementation leaders, Champions, External change agents, Innovation source, Evidence strength and quality |
| Collective action | Development process, Integration, confidence, Training, Leadership, Project manager, Accessibility of materials, Risk of missing patients, Electronic patient records, assessment, Burden of data collection | Adaptability, Networks and communication, Executing, Leadership engagement, Formally appointed internal implementation leaders, Available resources, Access to knowledge and information, Self-efficacy, Learning climate, Culture, Complexity, Goals and feedback, External policy and incentives |
| Reflexive monitoring | Regular meetings, Capturing data, Internal/external networks, Perceived value, Usefulness, Spread | Goals and feedback, Reflecting and evaluating, Networks and communication, Peer pressure, Complexity, Compatibility, Needs and resources of those served by the organisation, Tension for change, Knowledge and beliefs about the intervention, Evidence strength and quality, Relative advantage |
| Intervention performance | Partial implementation, Honour patient preferences, Electronic Patient Records | Executing, Reflecting and Evaluating, Compatibility |
| Relational restructuring | Integration, Respectful co-working, Collaborative development, Critical UCLP-Primrose roles, Team-based delivery | Cosmopolitanism, Networks and communications, Champions, Key stakeholders, External change agents, Opinion Leaders, Formally appointed internal implementation leaders |
| Normative restructuring | Champions, Access to practices / rooms, Wider transformation, Prevention rather than treating | Key stakeholders, Culture, Goals and feedback, Available resource, Executing, Opinion leaders |
| Sustainment (normalisation) | Additional ask, Partial implementation, Not core business, Impact of staff turnover, No or slow progress, Iterative | Reflecting and evaluating, Planning, Knowledge and beliefs about the innovation, Champions, Goals and Feedback |
