# Supplementary material for: Bridging the gap in the UK’s National Health Service integrated care systems: insights from a mixed methods implementation evaluation of UCLP-PRIMROSE, a care innovation to reduce physical health inequalities for people with severe mental illness
Source: BMJ Open. 2026 Jan 27;16(1):e105511. doi: 10.1136/bmjopen-2025-105511 (PMC12853453; doi:10.1136/bmjopen-2025-105511)
Supplement: Supplementary file 6 [file bmjopen-2025-105511.reviewer_comments.pdf]

PEER REVIEW HISTORY

BMJ Open publishes all reviews undertaken for accepted manuscripts. Reviewers are asked to complete a checklist review form and are provided with free text boxes to elaborate on their assessment. These free text comments are reproduced below.

ARTICLE DETAILS

Title (Provisional)

Bridging the gap in the UK's NHS integrated care systems: Insights from a mixed methods implementation evaluation of UCLP-PRIMROSE, a care innovation to reduce physical health inequalities for people with severe mental illness

Authors

Shaw, Philippa; Khurshid, Zuneera; Lamb, Danielle; Stevenson, Fiona A; Russell, Gregor; Hudson, Kristian; Rehill, Nirandeep; Copsey, Gemma; Kearney, Matt; Beveridge, Edward; Prenelle, Ian; Osborn, David

---

VERSION 1 - REVIEW

**Reviewer**	**1**
**Name**	**Arnautovska, Urska**
**Affiliation**	**The University of Queensland, Faculty of Medicine**
**Date**	**21-Jul-2025**
**COI**	**None**

This study reports findings of a mixed-methods evaluation of a novel intervention, UCLP-PRIMOSE, which aims to address the physical health comorbidity of people living with severe mental illness. There are several strengths of the study, including strong grounding in validated methodological frameworks, lived experience involvement across all stages of the research cycle, and a focus on improving an area of critical need for people with SMI, their physical health. The findings are innovative and highly insightful, with the implementation evaluation and the methodology potentially an exemplar model to other studies that will test the implementation of novel physical health-focused interventions within healthcare service settings serving people with SMI.

I have provided a few suggestions on how the manuscript could be improve for Authors' consideration below.

1. I suggest consistent wording regarding the use of terms "patients" and "participants" (eg line 253 refers to patients, while in the next paragraph, the term "participants" is used). Related to this, you may wish to consider using the term "people" rather than "patients" in

the Introduction, and possibly also in the title, since this is more in line with whole-person approach, which goes beyond considering people with SMI as primarily patients.

2. Abstract, line 35: Suggest adding the word "identify" before the processes, barriers and facilitators.

3. Introduction, line 110: Referring to the Lancet Commission is completely valid, however you may wish to back up this statement also with more recent evidence, eg a meta-analysis by Kisely et al on Breast cancer treatment disparities in SMI, published in Psycho-Oncology in 2023 (DOI 10.1002/pon.6120).

4. Introduction, lines 113-118: Given UCLP-PRIMROSE is a multidisciplinary approach, it might be relevant to note recent studies which used multidisciplinary approaches to improve physical health in people with SMI (eg DOI 10.1186/s12888-025-06830-3).

5. Methods: Please provide the targeted and achieved sample size in the main body text. If available, please also provide more details about the staff (eg position, discipline, clinical vs management role).

6. Results, line 253: The fact that no participant consented to the interview is intriguing, and contrary to the experience in our trials with patients within similar health service settings. Could the Authors elaborate on the requirements around the interview (eg was the interview required to be face-to-face, lasting a minimum of xx minutes etc). This information could be relevant to other studies, where participants' qualitative reflections would also be sought and could be integrated within the existing limitations section.

**Reviewer**	**2**
**Name**	**van Rensburg, André**
**Affiliation**	**University of KwaZulu-Natal, Centre for Rural Health**
**Date**	**30-Sep-2025**
**COI**	**None**

Thank you for the opportunity to contribute to peer reviewing the paper titled "Bridging the gap in the UK's NHS integrated care systems: Insights from a mixed methods implementation evaluation of UCLP-PRIMROSE, a care innovation to reduce physical health inequalities for patients with severe mental illness", submitted for consideration to BMJ Open. The topic is no doubt of high importance, and fits well within global and UK trajectories in health system reforms towards better integration and more people-centred systems of care. Process findings are incredibly important to publish, and there are well-known challenges in gathering and disseminating this kind of data. This being noted, several critiques emerged during review, which are summarised below:

- In general, the paper is written for an audience who are assumed to be fairly well-versed with UK health system dynamics, which is not the case for this journal. More contextualisation is required, by better describing UK reforms and goals, as well as how these interventions, policies and priorities fit within broader global trends. Interventions such as PRIMROSE no doubt has been in part made possible through advances in global health priorities, and this should be considered.

- On page 7, which Lancet Commission is referred to here?

- The claim that "There is ongoing debate around how to best deliver preventative and interventional care, fuelled by mixed findings across clinical trials examining lifestyle interventions for people with SMI (12–15)" requires more unpacking, e.g. what exactly is the debate about, and what does mixed findings mean?

- There is reference to a growing toolkit for SMI, but what does this include and where does PRIMROSE fit in?

- While there are descriptions of UCLPartners-PRIMROSE, a slightly more detailed description is warranted, especially with regards to the sites, and the people tasked to deliver specific tasks. Otherwise, it makes it difficult to follow the evaluation.

- The claim that "How to quickly translate and embed best practice into cross-sector care delivery has received relatively little attention" is simply inaccurate, given the substantial body of implementation science and knowledge translation evidence and frameworks, published across disciplines, especially in mental healthcare.

- With "We need to develop the current literature", is it meant to state that you need to build on existing evidence?

- The aim statement "We aimed to investigate whether UCLP-PRIMROSE could be set up and delivered as part of service transformation and identify the related facilitators, barriers and processes" requires more detail, especially clarifying what is meant by "service transformation".

- A mixed methods approach is purported, but what is the exact design of the study?

- Implementing integrated care across boundaries is a bit of an oxymoron, since integrated care is by definition working across boundaries.

- In the Methods section, more reasoning should be provided for decisions taken, e.g. why a period of 9 months was chosen for interviews to be undertaken, what informed the topic guides for the interview schedules, why was virtual interviews done and not another method?

- The reasoning behind the use of both NPT and CFIR is not well described, especially since CFIR contains much of NPT constructs. Also, it's not clear why the first version of CFIR was chosen and not the updated version. Further, while it can be appreciated that the authors wanted to make the findings more accessible by omitting framework constructs for

reporting, this choice carries a substantial challenge. It means that future meta-analyses would not be able to include this study as it would be too difficult to ascertain which constructs are being used. An audit trial or some kind of breakdown of which constructs were merged in the Findings section, and why, is perhaps needed. When using frameworks for analysis purposes and not engaging sufficiently with the theory, the robustness and structures that the frameworks are designed to provide is undermined.

- This was described, but the lack of service user voice/input should be seen as a major limitation, as should the lack of co-interpreting findings with participants through participatory workshops etc.

- Line 252-257, page 15, should be in the Methods

- Frequent mention is made of Integrated Care Systems, but it is unclear whether this is a policy (if so, what does it entail) or a construct.

- The terms “integration” and “integrated care” is used without due consideration of the multilayered nature of the concept, as it could include normative integration, organisational integration, programmatic integration etc.

- The theme “Developing stable relational and practical foundations within constrained contexts” seemed to focus more on leadership and championing programmes than resource use?

- Terms such as “breathing room” and “bandwidth” are not entirely clear, and more specific language should be applied.

- There needs to be much more engagement in the Discussion with a substantial body of evidence on the factors that influence intervention implementation, especially in terms of studies that have employed CFIR and NPT

VERSION 1 - AUTHOR RESPONSE

Reviewer: 1
Dr. Urska Arnautovska, The University of Queensland

Comments to the Author:
This study reports findings of a mixed-methods evaluation of a novel intervention, UCLP-PRIMOSE, which aims to address the physical health comorbidity of people living with severe mental illness. There are several strengths of the study, including strong grounding in validated methodological frameworks, lived experience involvement across all stages of the research cycle, and a focus on improving an area of critical need for people with SMI, their physical health. The findings are innovative and highly insightful, with the implementation evaluation and the methodology potentially an exemplar model to other studies that will test the implementation of novel physical health-focused interventions within healthcare service settings serving people with SMI.

I have provided a few suggestions on how the manuscript could be improve for Authors' consideration below.

I suggest consistent wording regarding the use of terms "patients" and "participants" (eg line 253 refers to patients, while in the next paragraph, the term "participants" is used). Related to this, you may wish to consider using the term "people" rather than "patients" in the Introduction, and possibly also in the title, since this is more in line with whole-person approach, which goes beyond considering people with SMI as primarily patients.	We agree that "people with SMI" is more in line with the whole-person approach. Therefore, we have taken this suggestion and updated in the title and introduction. In the methods and findings section we have kept "patients" where it is relevant – especially when referring to participants in the health setting which covers both patients and health professionals.
Abstract, line 35: Suggest adding the word "identify" before the processes, barriers and facilitators.	This has been added. On the revised copy this now appears on line 30.
Introduction, line 110: Referring to the Lancet Commission is completely valid, however you may wish to back up this statement also with more recent evidence, eg a meta-analysis by Kisely et al on Breast cancer treatment disparities in SMI, published in Psycho-Oncology in 2023 (DOI 10.1002/pon.6120).	Thank you for this recommendation. We have aimed to be purposeful with our choice of citations, focusing on physical health for people with SMI more generally, rather than additional conditions like cancer. However, we agree that bringing in more recent publication would be valuable. Since submission, two new Lancet Commissions have been published, one focused on "Holistic prevention and management of physical health side-effects of psychotropic medication" and the other "Implementing lifestyle interventions in mental health care". We have now added the latter into our introduction to reflect latest developments.
Introduction, lines 113-118: Given UCLP-PRIMROSE is a multidisciplinary approach, it might be relevant to note recent studies which used multidisciplinary approaches to improve physical health in people with SMI (eg DOI 10.1186/s12888-025-06830-3).	We have added in reference to multidisciplinary approaches including supporting this with the suggested citation where we have described effective components of lifestyle interventions for people with SMI (lines 120-122 on the revised manuscript), before moving into describing the shift to more joined up working in the NHS.

Methods: Please provide the targeted and achieved sample size in the main body text. If available, please also provide more details about the staff (eg position, discipline, clinical vs management role).	We have now added in our justification of sample size in the methods, under the subheading of 'sample size'.<br>We have also added in an aggregated list of self-defined staff roles in the table of demographic characteristics (Table 1).
Results, line 253: The fact that no participant consented to the interview is intriguing, and contrary to the experience in our trials with patients within similar health service settings. Could the Authors elaborate on the requirements around the interview (eg was the interview required to be face-to-face, lasting a minimum of xx minutes etc). This information could be relevant to other studies, where participants' qualitative reflections would also be sought and could be integrated within the existing limitations section.	We appreciate your interest in this. The interview itself was designed with flexibility and inclusivity in mind, with input from people with lived experience. Participants could choose between in-person, phone or online interviews, and had the option to be accompanied by a supportive other. We also worked with our patient and public involvement groups to develop both safeguarding protocols and options for reasonable adjustments.<br><br>As described in the paper – despite this, recruitment was challenging. As this was not a trial, there was no additional funding to support with delivery of UCLP-PRIMROSE or the research components. The administration involved with delivering UCLP-PRIMOSE was reported as a barrier, and research requirements were additional to delivery – some of the transferable lessons from this include building research capacity into routine care delivery.<br><br>Additionally, delays between consent and research contact also likely reduced participation. When the research team spoke to patients, there was hesitance about being recording or being unclear which aspects of care was UCLP-PRIMROSE. There was more success from other avenues of patient feedback, outside of the research, for example when asked directly by the care delivery team for feedback and Health Innovation Network collection of case study stories. This suggests that our research processes could be improved, for timeliness and additional consideration of ways to build rapport and trust. Moreover, our learnings include working with multiple stakeholders, to better distinguish requirements for trial and implementation studies - where possible simplifying the expected recruitment processes for the latter (lengthy information sheets and consent

	forms for example) whilst still meeting ethical standards.

Reviewer: 2
Dr. André van Rensburg, University of KwaZulu-Natal
Comments to the Author:
Thank you for the opportunity to contribute to peer reviewing the paper titled "Bridging the gap in the UK's NHS integrated care systems: Insights from a mixed methods implementation evaluation of UCLP-PRIMROSE, a care innovation to reduce physical health inequalities for patients with severe mental illness", submitted for consideration to BMJ Open. The topic is no doubt of high importance, and fits well within global and UK trajectories in health system reforms towards better integration and more people-centred systems of care. Process findings are incredibly important to publish, and there are well-known challenges in gathering and disseminating this kind of data.

In general, the paper is written for an audience who are assumed to be fairly well-versed with UK health system dynamics, which is not the case for this journal. More contextualisation is required, by better describing UK reforms and goals, as well as how these interventions, policies and priorities fit within broader global trends. Interventions such as PRIMROSE no doubt has been in part made possible through advances in global health priorities, and this should be considered.	Thank you for raising this, we have added some more context to help orientate a broader audience to the UK health system and related policy. This includes a definition of the Integrated Care System, as well as including more recent relevant updates to policy. For the latter we have added reference to Lord Darzi's report investigating the current state of the NHS, and included key publications regarding the current priorities and future direction of the NHS with the 10-year plan. This additional orientation has been added to our background section (lines 132-132 on the revised manuscript).<br><br>We agree that there is global attention in this area, which is discussed in the cited Lancet commissions, however, due to the unique context of the NHS at time of the research and this being key within our work, we have chosen to remain focused on this specific context.
On page 7, which Lancet Commission is referred to here?	The citation at the end of that sentence links to the reference "The Lancet Psychiatry Commission: a blueprint for protecting physical health in people with mental illness. Lancet Psychiatry". To make this clearer we have now added the full name of the commission directly into the sentence (lines 114 and 115 on the revised manuscript).
The claim that "There is ongoing debate around how to best	We first clarified this statement – mixed findings related to clinical outcomes in trials and

deliver preventative and interventional care, fuelled by mixed findings across clinical trials examining lifestyle interventions for people with SMI (12–15)" requires more unpacking, e.g. what exactly is the debate about, and what does mixed findings mean?	systematic reviews with meta-analysis, with studies showing variation in physical health outcomes.<br><br>However, we have now replaced this sentence due to updated evidence since submission, particularly the latest Lancet Commission on implementing lifestyle interventions. This offers additional clarity of what best practice should look like and more certainty on the positive impact of such intervention based on the wealth of research more recently published, fuelled by the 2019 Lancet Commission.
There is reference to a growing toolkit for SMI, but what does this include and where does PRIMROSE fit in?	Whilst it is not within scope of this paper to discuss all of the different approaches available (this has been explored in the cited Lancet Commission), we agree there is benefit in adding a sentence on additionally positioning PRIMROSE.<br><br>We have therefore added two sentences to show where UCLP-PRIMROSE fits in, highlighting some of the features relative to what is explored in the Lancet Commission into implementation of interventions for the physical health of people with SMI (lines 147-152 in the revised manuscript).
While there are descriptions of UCLPartners-PRIMROSE, a slightly more detailed description is warranted, especially with regards to the sites, and the people tasked to deliver specific tasks. Otherwise, it makes it difficult to follow the evaluation.	When considering the target wordcount of this journal it was necessary to be brief in the description of UCLPartners-PRIMROSE within the main body of the article. In addition to this, our figures provide further information: the pathway map (Figure 1) breaks down the tasks at each stage of delivery, and Figure 3 presents the different health professionals who were involved at each stage of the pathway across sites. Moreover, in line with the journal recommendations, we supplemented this the TIDieR checklist (Additional File 1). This Additional File is comprehensive – it includes full details of core components, training, delivery methods and information on who could be involved with the delivery of specific tasks, as well as the sites involved.<br><br>We believe we have been comprehensive through use of combined description, figures, additional files, and citing to previous publications. Therefore, we have not expended our description other than clarifying the signposting to Additional File 1 (See

	Additional File 1 for further details on the clinical model).
The claim that "How to quickly translate and embed best practice into cross-sector care delivery has received relatively little attention" is simply inaccurate, given the substantial body of implementation science and knowledge translation evidence and frameworks, published across disciplines, especially in mental healthcare.	Whilst the field of implementation science is rapidly developing, its application to innovations which are integrated in physical health for SMI and also span primary care, secondary care, and the voluntary sector, has not had substantial attention. Moreover, UK Integrated Care Systems were still in their infancy at the time of study, and therefore a novel setting for the application of implementation science.<br>We have reordered the information in the manuscript to better articulate this point.
With "We need to develop the current literature", is it meant to state that you need to build on existing evidence?	Yes – we have updated this with the preferred language (line 133 on the revised manuscript).
The aim statement "We aimed to investigate whether UCLP-PRIMROSE could be set up and delivered as part of service transformation and identify the related facilitators, barriers and processes" requires more detail, especially clarifying what is meant by "service transformation".	We have clarified this within the manuscript. By service transformation we are referring to changes in care delivery / improvement that happen outside of research-controlled environments like clinical trials. In our case, UCLP-PRIMROSE was being delivered as a clinical initiative, with research focused on the implementation – observing the uptake, processes, barriers and facilitators.
A mixed methods approach is purported, but what is the exact design of the study?	Thank you for catching this, we have now defined our design as "convergent parallel design" on line 159 of the revised manuscript.
Implementing integrated care across boundaries is a bit of an oxymoron, since integrated care is by definition working across boundaries.	Thank you for prompting clarification here. We describe UCLPartners-PRIMROSE as an integrated approach to care as it brings together mental and physical health, whilst considering wider determinants of health (we use integrated over holistic due to preferences of our patient and public involvement panel). Then there is an additional level of integration, which is across care boundaries (primary and secondary care, with the voluntary sector).<br><br>We have clarified this by adding "integrated mental and physical health pathway across care boundaries" on lines 160 & 161 of the revised manuscript.

In the Methods section, more reasoning should be provided for decisions taken, e.g. why a period of 9 months was chosen for interviews to be undertaken, what informed the topic guides for the interview schedules, why was virtual interviews done and not another method?	As with several of our other responses, the word count necessitated us to be brief where possible.<br><br>We have additionally clarified the timeline on lines 173 to 180 of the revised manuscript (allowing for sufficient time to start delivery, whilst also accommodating for site preferences for this to start with the new QOF year and our overall research timeline).<br><br>We have also added clarity on mode of interview – the option was given for online, phone, or in-person (with all but one selecting online – lines 181-183) – and the topic guide informed by CFIR (described on lines 184-190 in the revised manuscript).
The reasoning behind the use of both NPT and CFIR is not well described, especially since CFIR contains much of NPT constructs. Also, it's not clear why the first version of CFIR was chosen and not the updated version. Further, while it can be appreciated that the authors wanted to make the findings more accessible by omitting framework constructs for reporting, this choice carries a substantial challenge. It means that future meta-analyses would not be able to include this study as it would be too difficult to ascertain which constructs are being used. An audit trial or some kind of breakdown of which constructs were merged in the Findings section, and why, is perhaps needed. When using frameworks for analysis purposes and not engaging sufficiently with the theory, the robustness and structures that the frameworks are designed to provide is undermined.	Thank you for raising this, we have added a section titled 'rationale' (from line 234) within the *Data analysis* section to clarify our rationale and to make the theoretical positioning more explicit.<br><br>While there is some conceptual overlap between CFIR and NPT, we see them as mutually supportive – CFIR is a determinant framework whereas NPT allows us to focus on the social processes of implementation. Using both was proposed to support us capture a more rounded understanding of implementation.<br><br>We have also clarified that the original version of CFIR was applied because data collection and early coding commenced prior to the release of CFIR 2.0. We now note this explicitly in the manuscript (lines 255-257 in the manuscript). In our subsequent work (discussed below) we have mapped our findings across from the original CFIR to CFIR 2.0.<br><br>We agree that additional information demonstrating the robustness of application would be beneficial. We have therefore referenced and included Additional File 5, which provides a table of the mapping across CFIR, reflexive thematic analysis codes, and NPT.

	The additional points raised are addressed in our subsequent manuscripts which are written for audiences more familiar with implementation science.<br>The first is a detailed reporting of the CFIR findings, exploring these between locations as well as exploring the meaning of this related to micro, macro and meso structures. This paper has been submitted to a journal and is under review.<br><br>The second additional publication is in progress and focusses on our methodology. We present UCLP-PRIMROSE and the findings from across the research in an Implementation Research Logic Model (IRLM). We then use the IRLM to critically reflect on the use of our different approaches (including NPT and CFIR) and the value and difference they made to our work.<br><br>We have signposted to future publications in our strengths and limitation section.
This was described, but the lack of service user voice/input should be seen as a major limitation, as should the lack of co-interpreting findings with participants through participatory workshops etc.	We agree this is an important point, which is why it has been highlighted in our manuscript.<br><br>For the second point however, throughout our research we have worked closely with a patient and public involvement group, and our collaboration included discussions of findings, their implication and then co-creation of outputs (the group particularly wanted to focus on developing outputs to share the findings with the public). We have clarified this in our patient and public involvement section.
Line 252-257, page 15, should be in the Methods	We have taken this paragraph and broken this up into the relevant methods sections.
Frequent mention is made of Integrated Care Systems, but it is unclear whether this is a policy (if so, what does it entail) or a construct.	We have now added a description of UK Integrated Care Systems within our background sections (line 123-128) to better contextualise for readers.
The terms “integration” and “integrated care” is used without due consideration of the multilayered nature of the concept, as it could include normative integration,	Where possible we have further specified our use of integration, such as “system integration” or used alternative terms when appropriate like “collaboration”

organisational integration, programmatic integration etc.	
The theme “Developing stable relational and practical foundations within constrained contexts” seemed to focus more on leadership and championing programmes than resource use?	Our research showed these were linked, as described in this theme. Leadership and champions are both important elements of this section of the manuscript (identified with CFIR as an enabler when present). We have drawn this together with the processes these individuals can support (from an NPT mechanism perspective), where resources are particularly relevant.<br><br>So whilst tools like lesson plans and implementation meetings were available (CFIR access to knowledge, and networks and communication) without ringfenced and prioritised resourcing (time, funding, space) some opportunities for embedding (NPT – collective action and reflexive monitoring for example) were limited.
Terms such as “breathing room” and “bandwidth” are not entirely clear, and more specific language should be applied.	We have updated our use of breathing room to capacity, and bandwidth to cognitive capacity and/or time and cognitive capacity.
There needs to be much more engagement in the Discussion with a substantial body of evidence on the factors that influence intervention implementation, especially in terms of studies that have employed CFIR and NPT	For this paper we have purposefully grounded both the findings and the discussion in practical and accessible exploration of UCLP-PRIMROSE implementation within the current context.<br><br>As mentioned above we have in progress papers which delve into the methodological side of our work. We have added into our strengths and limitations section that the aim of this paper was to report on implementation, and our methodological engagement with CFIR and NPT will be the topic of further papers.

VERSION 2 - REVIEW

Reviewer 1

Name Arnautovska, Urska

Affiliation The University of Queensland, Faculty of Medicine

Date 25-Nov-2025

COI

The Authors have addressed the reviewers' comments thoroughly and also strengthened the paper by supporting their statements with a few recent relevant publications such as Lancet Commission on the implementation of lifestyle interventions.

Overall, I believe the paper will make an important contribution to the field and inform other novel interventions designed to improve health outcomes in people living with severe mental illness.
